# Miniaturized Frequency Selective Surface for 6G Communication

**DOI:** 10.3390/mi13030427

**Published:** 2022-03-10

**Authors:** Jiufu Ruan, Zifan Meng, Ruizhi Zou, Fei Cai, Shengmin Pan

**Affiliations:** 1 Special Display and Imaging Technology Innovation Center of Anhui Province, National Engineering Laboratory of Special Display Technology, Academy of Opto-Electric Technology, Hefei University of Technology, Hefei 230009, China; jfruan@hfut.edu.cn (J.R.); zfmeng@mail.hfut.edu.cn (Z.M.); rzzou@mail.hfut.edu.cn (R.Z.); 2 Anhui Province Key Laboratory of Measuring Theory and Precision Instrument, School of Instrument Science and Opto-electronics Engineering, Hefei University of Technology, Hefei 230009, China; caifei@hfut.edu.cn; 3 Institute of Plasma Physics, Chinese Academy of Sciences, Hefei 230031, China

**Keywords:** terahertz, 6G communication, circuit model, microfabrication

## Abstract

A single-layer, quartz-supported frequency selective surface (FSS) with a gear-shaped metallic array is proposed for 6G communication. Full-wave simulation, along with the method of equivalent circuit, is applied to investigate the transmission characteristics, while the distributions of surface current distribution, as well as electric field and magnetic fields, are studied to further interpret the transmission mechanism. The simulation indicates that the resonant frequency of 131 GHz with an attenuation of −40 dB can be obtained and the relative bandwidth approximates to 12%. The transmission response of the fabricated FSS prototype is measured using the free space measurement setup. The measured results show a good agreement with the simulated ones, which demonstrates the reliability of the design and fabrication. The proposed FSS with the advantages of simple structure, low cost, easy fabrication, and integration can be applied in enhancing the communication performance and anti-interference ability in the future 6G communication system.

## 1. Introduction

With the commercial application of 5G communication technology, 6G [1] technology has been under study for greater data rate and less latency, in which the ultra-wide frequency domain 73–140 GHz and 1–10 THz can be employed [2]. Nevertheless, considering the realization of power source and system integration, the most promising candidate for 6G communication is the frequency range of 73–140 GHz. Consequently, various kinds of devices for the next generation communication, such as absorbers [3], antennas [4,5,6], filters [7,8], lens [9], are in urgent demand and these devices must meet the requirements of low-cost, miniaturization and simple-structure for compact and high integration systems.

Frequency selective surface (FSS) [10,11,12] is a solution to the aforementioned requirements [13]. It is known that FSS is a kind of periodic structure that has been adopted in a variety of applications including filter, antenna reflector and radome, absorber, electromagnetic (EM) shielding ranging from microwave to terahertz frequencies. Hence, FSS has been a research hotspot for the past half-century; there are numerous FSSs at the band of 1–60 GHz and also many articles reporting the FSSs at the band of 20~100 GHz and high-terahertz frequencies beyond 200 GHz [14,15,16,17,18,19]. However, to the best of our knowledge, only several FSSs of 100 GHz~200 GHz have been reported to date [20,21,22,23]. Admittedly, these FSSs have some performance advantages for their application fields. For example, the designed FSS in [20] has better polarization stability due to the use of hexagon cavity. Of course, its structure and fabrication are a little complex just because of this. The FSS reported in [21] can provide a flat passband. But the eight-layer structure makes its fabrication difficult. The proposed FSS in [22] can afford quad-band frequency response and it is limited by a large thickness in many applications. The FSS in [23] is simple in structure. Meanwhile, its performance is a little weak. Hence, there is a need to make a balance between structure complexity, thickness, and fabrication.

In this paper, a compact FSS with a resonant frequency of 131 GHz and the stopband bandwidth of 16 GHz is presented. The FSS can excellently play the filtering role at the frequency range most likely to be adopted for 6G communication. The FSS has the simple structure of a single-layer, gear-shaped, metal arrays deposited on quartz glass, which makes it low-cost and easy fabrication.

## 2. Structure, Analysis, and Discussion

Since the miniaturized FSS is preferable due to the fact that the same-sized surface can contain more unit cells, many methods have been employed to miniaturize the FSS unit cells. No matter which method is used, the principle of these methods is to increase the electrical length of unit cells with the same physical area. However, this could lead to a complex FSS structure. Therefore, the design criteria of the FSS in this work are to realize the miniaturization and a single-layer metallic array without increasing the complexity of the structure and degrading the filtering performance. Based on this design principle, the circular loop, which is the conventional and simple FSS structure, is chosen as the basic configuration in this work. To further accomplish the miniaturization and obtain higher performance, the method of enhancing the inductance and the capacitance can be adopted by adding four teeth to the loop and forming a gear-shaped structure. With the same FSS area, the additional teeth play the role of increasing the electrical length without significantly complicating the structure. Thus the miniaturization of the FSS can be fulfilled using the single-layer metallic array and the simple pattern.

**Figure 1:**
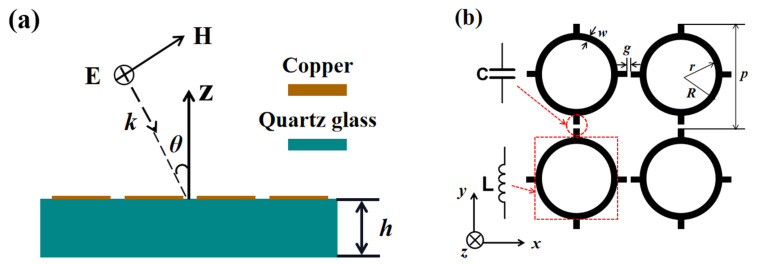
(**a**) Side view of the proposed FSS; (**b**) geometry of metallic array.

The geometry of the proposed FSS is shown in Figure 1a. It can be observed that the FSS was composed of a metallic array with a thickness of 600 nm patterned on the surface of a dielectric substrate with a thickness of 1 mm. The copper with an electrical conductivity *σ* = 5.8 × 10^7^ S/m and the quartz glass with a relative permittivity of 4.82 and loss tangent of 0.1 were chosen as the material of metallic array and dielectric layer, respectively. Figure 1b shows the gear-shaped structure of the metallic array. As illustrated in Figure 1b, the period of the metallic array is denoted as *p*, and the inner and the outer radius of the circular loop are labeled as *r* and *R*, respectively. Accordingly, the loop width is *w = R-r*. The gap between the two adjacent unit cells is *g*. The proposed single-layer FSS is simulated and optimized using CST Microwave Studio software. These parameters were as follows: *p* = 499 μm, *R* = 220 μm, *r* = 200 μm, *w* = 20 μm, *g* = 2 μm.

**Figure 2:**
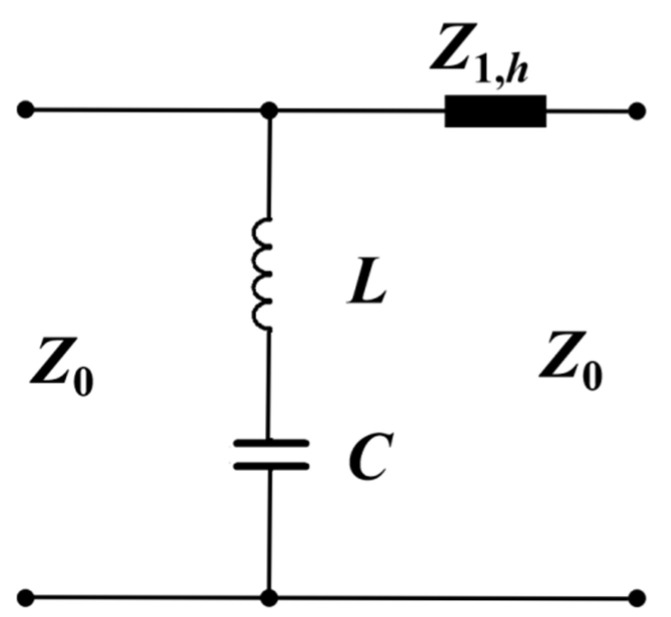
Equivalent circuit model of the proposed FSS.

The equivalent circuit of the proposed structure is used to interpret the physical mechanism of the operation principle. As depicted in Figure 1b, the gaps between the two teeth of the two adjacent unit cells can be represented by a capacitor, while the circular loop can be regarded as an inductor. Then the corresponding equivalent circuit of the FSS is a simple resonant circuit comprised of an inductor in series with a capacitor, shown as Figure 2. *Z_0_* with the value of 377 Ω is the impedance of free space. *L* and *C* are the equivalent inductance and capacitance of the FSS, respectively. The transmission line *Z_1,h_* represents the substrate. The first-order approximation of the inductance *L* and capacitance *C* in Figure 2 can be described simply as [24,25].

(1) $$L=\mu \frac{p}{2\pi }\ln\left(\frac{1}{\sin\frac{\pi w}{2p}}\right)$$

(2) $$C=\varepsilon_{0}\varepsilon_{eff}\frac{2\pi }{w}\ln\left(\frac{1}{\sin\frac{\pi g}{2w}}\right)$$

(3) $$\varepsilon_{eff}=\frac{\varepsilon_{r}+1}{2}$$

where *p* is the period of the metallic array, *w* is the width of the circular loop, *g* is the gap between the adjacent unit cells, *ε_0_* is vacuum permittivity, *ε_r_* is the relative permittivity of the glass substrate, *ε_eff_* is the effective permittivity of the substrate, *µ* is the effective permeability of the structure. It must be noted that the equivalent circuit is only valid for normal incident waves. From the Equations (1) and (2), it can be determined that in this design of the FSS, adding four teeth to the loop can enhance the inductance and the capacitance by increasing the electrical length and decreasing the gap of the adjacent unit cells. The resonant frequency can be calculated as

(4) $$f=\frac{1}{2\pi \sqrt{LC}}$$

**Figure 3:**
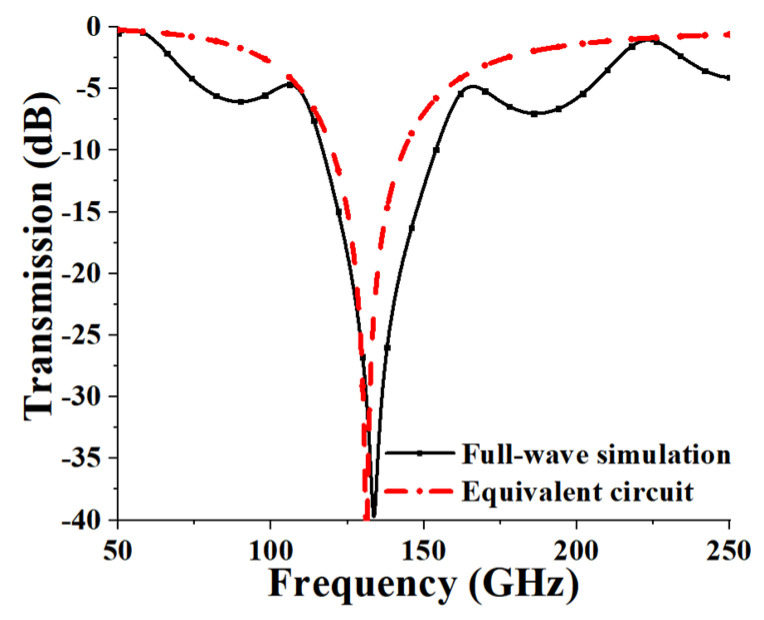
Transmission response under normal incidence: full-wave simulation results (solid line) and equivalent circuit analysis (dashed line).

The frequency characteristics of the optimized FSS using full-wave simulation as well as the equivalent circuit are shown in Figure 3. First, the results obtained from full-wave simulation are analyzed. The resonant frequency of 131 GHz with a deep attenuation up to −40 dB can be realized. The stopband bandwidth for −20 dB insert loss reference was 16 GHz and the relative bandwidth is 12%. As can be calculated using the Equations (1) and (2), *L* = 0.0566 nH and *C* = 0.02599 pF. By taking the two values into Equation (4), the resonant frequency of 131.22 GHz can be obtained, which is equal to the result of resonant frequency from full-wave simulation. Furthermore, the frequency range over the stopband calculated from the equivalent circuit is consistent with that of full-wave simulation. In general, the simulated results of full-wave simulation agree with the calculated ones of the equivalent circuit, which demonstrates the validity of the circuit analysis.

**Figure 4:**
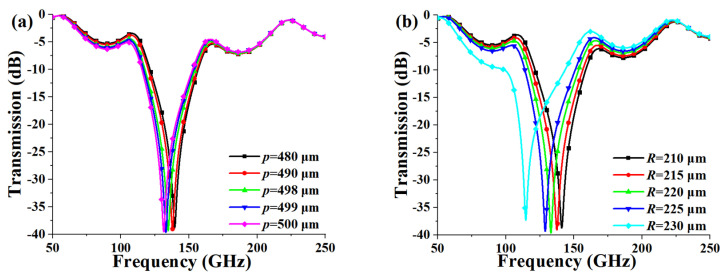

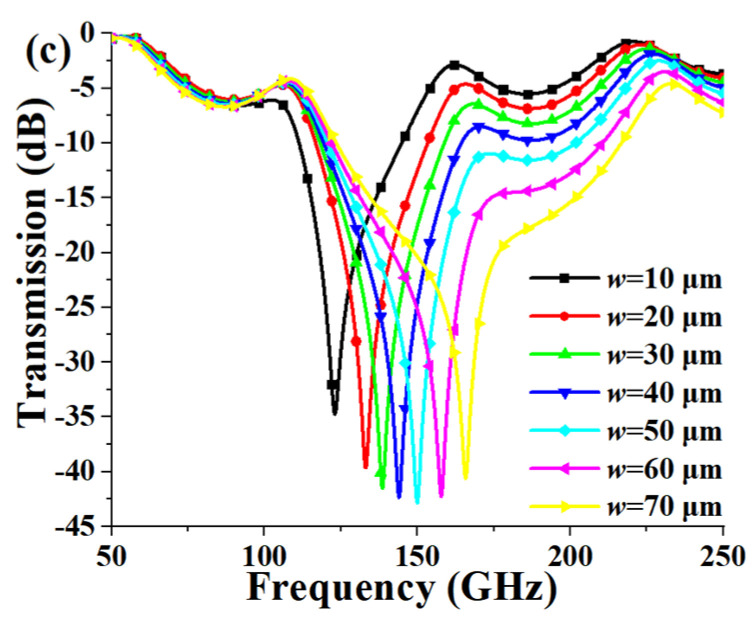
Influence of the structural parameters: (**a**) period *p*, (**b**) outer radius *R*, (**c**) width of the circular loop *w* on transmission response.

The next parameter study was performed to further interpret the operation principle of the FSS. As can be seen from Figure 1b, the structural parameters included the period *p*, the outer and inner radius of the loop *R*, *r*, the width of the loop *w,* and the gap *g* between the adjacent unit cells. Since the period *p* is related to the gap *g* and the inner radius *r* is connected with the outer radius *R* and the width of the loop *w*, it was just needed to investigate the influence of the three parameters of *p*, *R,* and *w* on transmission response, which is plotted in Figure 4. It can be observed from Figure 4a that the resonant frequency shifts to lower frequency as the period *p* increases. The red shift of the resonant frequency can be explained using the equivalent circuit. The gap *g* becomes smaller with the increase of *p*, and the equivalent capacitance *C* increases according to Equation (2). So the resonant frequency gets to a lower frequency, which can be known from Equation (4). As shown in Figure 4b, the resonant frequency also shifts to lower frequency with the increase of the outer radius *R*. It can also be interpreted that based on Equation (1), the equivalent inductance *L* grows larger due to the increase of the electrical length caused by the rise of *R*. As a result, the resonant frequency shifts to lower frequency according to Equation (3). Figure 4c illustrates the influence of the loop width on transmission response. It can be seen that as the loop width *w* increases, the bandwidth grows wider, which can be adopted to adjust the bandwidth.

**Figure 5:**
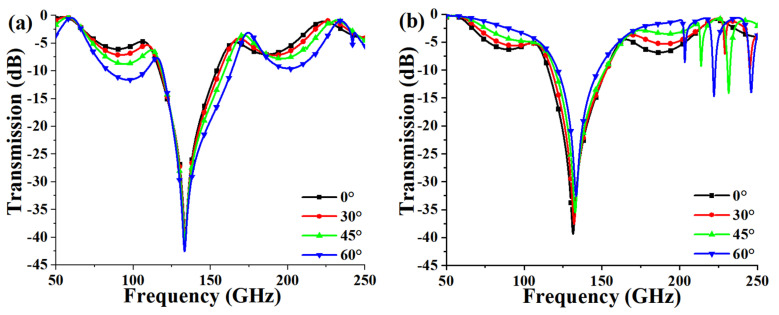
Transmission response under different incident angles for (**a**) TE polarization and (**b**) TM polarization.

Due to the obvious symmetry of the structure, the proposed FSS is insensitive to polarization, which is not studied in detail here. Since the incident waves could be from various directions, the angle-insensitive devices are more expectable. Therefore, Figure 5 depicts the transmission response under different incident angles for TE mode and TM mode. It can be observed that the frequency response of the FSS is stable for different incident angles, and the bandstop transmission response is basically unchanged at incident angles below 60° for −20 dB reference, which indicates that the structure can be adopted to more complex applications due to the incident-angle insensitivity.

**Figure 6:**
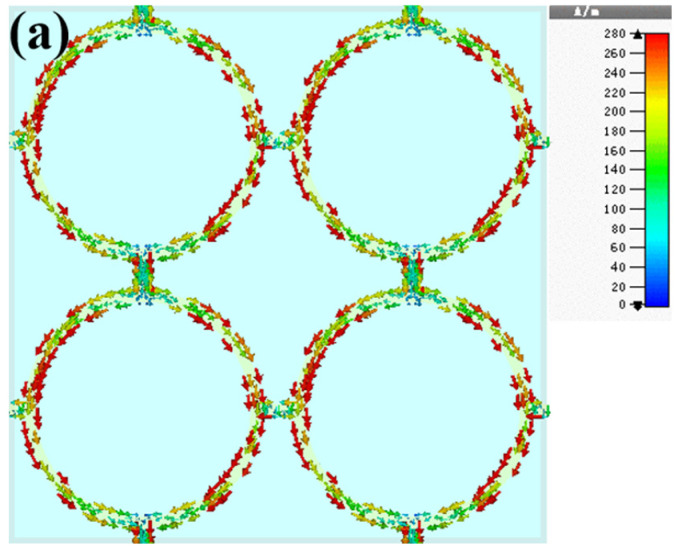

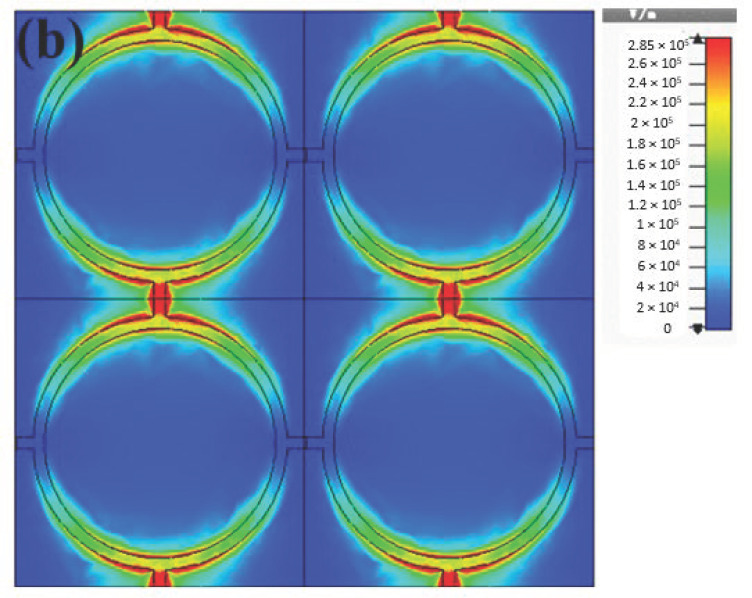
Distributions of (**a**) surface current and (**b**) electric field at resonant frequency.

To give further physical insights into the resonance mechanism of the proposed FSS, the distributions of the surface current and electric field at the resonant frequency were investigated and the results are depicted in Figure 6. As illustrated in Figure 6a, the currents flow from the top to the bottom along the two sides of the loop. As a result, the positive charge gathers at the bottom of each unit cell, while the negative charge accumulates at the top. Thus, as shown in Figure 6b, a strong electric field generates between the two adjacent unit cells, thereby producing a strong electric resonance.

## 3. Fabrication and Measurement

To validate the robustness of the design, the glass-supported FSS was fabricated using microfabrication technology. The detailed steps in the fabrication process are introduced as follows. First, the quartz glass was ultrasonic-washed by acetone, ethanol, and deionized water in succession and blow-dried with pure nitrogen flow. Then the clean glass was placed on the roof of the evaporation chamber and the high-purity copper with a mass of 1 g was put at the tungsten crucible located at the bottom of the chamber. When a vacuum degree below 10-5 Torr was reached, the current was applied for evaporation until all the copper was exhausted, and as a result, a copper layer with a thickness of about 600 nm was deposited on the surface of the quartz glass. Next, the positive photoresist AZ4562 with low viscosity was spin-coated with the spin speed of 3500 rpm for 60 s to form a 10 μm-thick layer, followed by baking at 60 °C and 100 °C for 10 min, respectively, to volatilize the solvent in the photoresist thoroughly. Subsequently, the maskless lithography technology was performed for exposure and then the developer AZ400K was used to develop the pattern. Finally, low-cost wet etching was employed for the removal of the redundant copper beyond the gear-shaped pattern.

**Figure 7:**
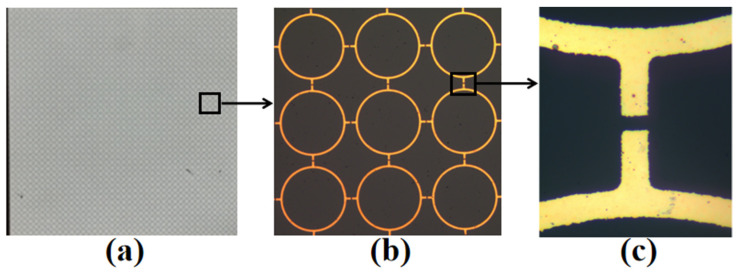
(**a**) Fabricated prototype of the proposed FSS; (**b**) Microscopic image of 3 × 3 array; (**c**) Zoom picture for highlighting the gap.

It is noted that the finite surface should contain a large number of FSS elements in order to observe the desired frequency response. As shown in Figure 7, the fabricated prototype is 20 mm × 20 mm in size and contains 40 × 40 unit cells.

**Figure 8:**
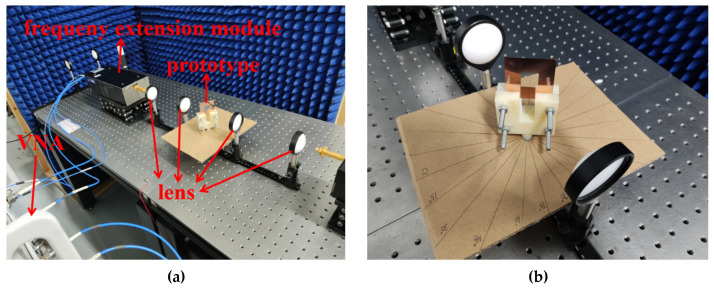
(**a**) Free space measure setup used for measurement of the proposed FSS. (**b**) Arrangement of prototype for measurement under different angles.

The transmission response of the fabricated prototype was measured using the free-space measurement setup. The free-space measurement setup shown in Figure 8 includes an Agilent vector network analyzer (VNA), the two frequency extending modules, two standard horns, and four lenses. Each end of the two frequency extending modules was connected to one port of Agilent VNA and the other end was connected to the standard horn. The tested prototype mounted in a holder was put in the middle of the two horns. The centers of horns, lenses, and tested prototype must be adjusted in a straight line on the same horizontal plane. The lens array, composed of four lenses, was adopted to collimate the radiated wave beam from the horn antenna, thereby making the electromagnetic (EM) wave incident on the FSS be a plane wave, which was the same as that in the simulation. The measurement system is calibrated using the free-space thru response. To be specific, transmission response was measured and recorded as a calibration reference without the FSS prototype first. Then the prototype was placed between the two horns and the transmission response was tested and recorded. Finally, the measured response of the prototype was the value of the latter minus the former.

**Figure 9:**
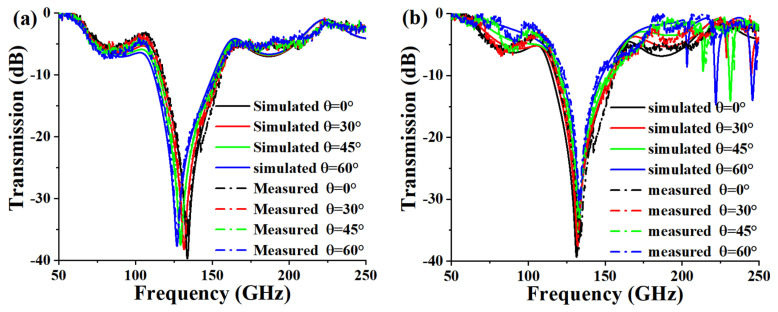
Comparison of measured and simulated results for different incident angle. (**a**) TE mode. (**b**) TM mode.

Figure 9 depicts the comparison of the measured results and the simulated ones at different incident angles under TE and TM modes. It can be seen that the measured results are basically consistent with the simulated ones, which proves the robustness of the design and the reliability of the fabrication.

Table 1 shows a comparison of the proposed FSS with the reported FSSs beyond 100 GHz in terms of bandwidth, the maximum angle of incidence, and transmission at the resonant frequency. It can be seen that the proposed FSS had a better performance of bandwidth and incident-angle stability. 

## 4. Conclusions

In this paper, a single-layered FSS is proposed for 6G communication, which consists of a 600 nm thick layer of gear-shaped metallic array deposited on 1 mm thick quartz glass. The simulation results reveal that the FSS exhibits excellent bandstop filtering, the resonant frequency of 131 GHz with −40 dB attenuation can be obtained, and the relative bandwidth is 12%. In addition, the proposed FSS is insensitive to polarization and incident angles. The prototype of the FSS was fabricated and the transmission response was experimentally obtained with free space measurement. The measured results are basically consistent with the simulated ones, verifying the reliability of the design and the fabrication. Endowed by the simplest structure, the proposed FSS with the advantages of low cost as well as easy fabrication and integration is a proper choice for bandstop filtering applications, which makes it applicable in enhancing communication performance and anti-interference ability in the future 6G communication systems.

## Figures and Tables

**Table 1:** Comparison of the proposed FSS with the reported FSSs operating beyond 100 GHz.

FSSs in Reference	Type	Bandwidth/GHz	Maximum Angle of Incidence/°	Transmittance at the Notch Frequency/dB
[21]	Bandpass	10	15	0.28
[22]	Bandpass	10	Not reported	1
[23]	Bandstop	20	Not reported	40
This work	Bandstop	37	60	39
